# Wealth inequalities in nutritional status among the tribal under-5 children in India: A temporal trend analysis using NFHS data of Jharkhand and Odisha states - 2006-21

**DOI:** 10.1016/j.dialog.2023.100135

**Published:** 2023-04-23

**Authors:** S. Rekha, P. Shirisha, V.R. Muraleedharan, Girija Vaidyanathan, Umakant Dash

**Affiliations:** a Department of Humanities and Social Sciences (DoHSS), Indian Institute of Technology (IIT), Madras, India; b Institute of Rural Management Anand, Gujarat, India

**Keywords:** Malnutrition, stunting, wasting, Underweight, Wealth inequalities, Tribal children, Jharkhand, Odisha, Under 5 children, Maternal and child health

## Abstract

**Background:**

Undernutrition remains a major public health concern in India, especially among children belonging to the Scheduled Tribes (ST). In this study, we analyse wealth inequalities in nutritional outcomes within ST communities in two tribal-dominated states of India, namely, Odisha and Jharkhand. The study also compares the trends in nutrition outcomes between ST and Non-ST children in these states.

**Methods:**

We have conducted a trend analysis of the prevalence and inequalities in the nutritional indicators among ST children under age five using unit-level data of the National Family Health Survey (NFHS) [NFHS-3(2005–06),4 (2015–16) and 5(2019–2021)]. Wealth-related inequalities were analysed using the Slope Index of Inequality (SII), which measures absolute inequality, and the relative Concentration Index (CIX), which measures relative inequality. We have also analysed the correlation between Antenatal Care (ANC) visits and nutritional indicators using the Pearson Correlation test.

**Results:**

The trend analysis shows that the prevalence of undernutrition remains higher among ST children in India as compared to Non-ST children between NFHS-3 (2005–06) and NFHS-5 (2019–2020) in Jharkhand and Odisha. The SII and CIX values show that statistically significant inequalities in stunting and underweight exist among children belonging to various wealth quintiles within the ST category in both states. Wasting is found to be significantly prevalent across all wealth quintiles. Also, we found a negative association between ANC visits and all three nutritional indicators.

**Interpretation:**

Our study highlights the importance of monitoring both the absolute and relative wealth inequalities in nutritional outcomes. This is due to the fact that while inequalities across groups may reduce, the prevalence of poor nutritional outcomes may increase among certain groups. Such observations, therefore, will enable policymakers to focus further on those groups and devise appropriate interventions.

## 1 Introduction

Malnutrition is a major public health concern in low-and-middle-income countries (LMICs) [[1], [2], [3], [4], [5], [6]]. Nearly 165 million children, under the age of five, are malnourished in LMICs [4]. The proportion of undernourished children in 2017 accounted for 20.4% in Africa, 11.4% in certain regions in Asia, and 6.1% in Latin America. Evidence indicates an increase in undernourishment and severe food insecurity in almost all the regions of Africa and South America, whereas, the undernourishment situation in Asia is observed to be stable [7]. According to more detailed disaggregated data, in poor and lower-middle income nations, where the average daily income is less than $2.80 per person per day, 37.8 million children are impacted by stunting. Another 101.1 million children are stunted in lower-middle-income nations, where the daily income per person is less than $11. Both the number of people affected (37.0 million) and the highest prevalence of wasting (11.5%) occur in lower-middle-income countries and are lowest (0.5 million and 0.7%, respectively) in high-income countries [1].

In India, every year, nearly 7·6 million children die before age five, and the largest deaths arise from preventable or treatable illnesses [8,9]. 35% of these deaths result from malnutrition. A malnourished child is ten times more likely to die than a well-nourished child from a preventable cause [9]. The nutritional status of under-5 children in India, estimated using the anthropometric data on height and weight collected in the fifth round of the National Family Health Survey (NFHS-5; 2019–20), indicates that 35·4% are stunted (height-for-age deficit), 32·08% are underweight (weight-for-age deficit), and 19·35% are wasted (weight-for-height deficit) [10] India's progress in childhood nutrition status does not commensurate with its economic progress, and it is worse off than most Sub-Saharan nations [11] This paradoxical situation in India, as in most other South-Asian countries, is called the ‘Asian Enigma’.

The nutritional situation among the children of Scheduled Tribes (ST) in the country is worse when compared to their non-ST counterparts [12]**.** The ST population in India is 104 million (as per the Census of India 2011), which accounts for 8.6% of the country's total population [13]. According to Article 366/342 of the constitution of India, the criterion followed for the specification of a community as scheduled tribes are indications of primitive traits, distinctive culture, geographical isolation, shyness of contact with the community at large and backwardness [14].

The lives of the tribal population in India have been largely impacted by various economic, political, social, and cultural changes. It is important to situate and assess the development status of the tribal communities before the time they have been incorporated into the current economic and political structure, as the change in the wider social structure has been tied to the loss of their life-supporting system, which includes land, forest, water and other natural resources. This adversely impacted the living and health conditions of the tribal population in India [15]. Thereby, over the years, the dietary practices of tribal groups have changed, adversely affecting the dietary diversity due to lack of access to the forest, loss of livelihoods, migration, acculturation and the growing reliance on the public distribution system [16] Inadequate intake of micro-nutrient-rich foods, like green vegetables, and lack of nutritional awareness among the tribal women, are causing significant health consequences, including under-nutrition and anaemia [17] Their daily nutrient intake has come down to 50 g/CU/day between the second (1988–90) and third (2008–09) surveys of the National Nutrition Bureau (NNB) [16]. Nevertheless, there has been an improvement in nutritional indicators of the ST population over the years. This improvement is assumed to result from reduced physical labour, better access to safe drinking water, improved socio-economic conditions and better utilization of health care services [16].

India has one of the highest levels of wealth inequality in the world. In India, the top decile owns a larger proportion of wealth, with over 65% of the total country's wealth [18]. These persistent economic inequalities have led to a larger concentration of anaemia, stunting, wasting, and underweight among the children belonging to the poorest wealth quintile [19]. Chancel and Piketty (2017) estimated that the top 0.1% of earners captured a higher share of total growth than the bottom 50% (12% vs 11%), and the top 1% received a higher share of total growth than the middle 40% (29% vs 23%) [20]. Studies on caste stratification and wealth inequality have observed that socially disadvantaged communities, particularly Scheduled Tribes and Scheduled Castes (STs/SCs), have lower wealth than other caste categories. Also, there is an emergence of a rich “creamy layer” or relatively well-off groups within these disadvantaged communities [21]. The existing social stratification and class formation among the tribal population was structured based on their history, level of economic development, nature of the colonial impact, and exposure to modern forces of social transformation [22].

Most studies treat tribal communities as an egalitarian group with a collective conscience, and, fail to capture and address the question of differentiation and inequality among them. Even though since Independence, various publicly financed initiatives have significantly improved the overall living conditions of the Tribal population across India, the inequality across all social classes and within ST communities continues [11,22]. Such inequalities are also reflected in the health status of the ST population. It is essential to have an understanding of the inequalities in health status within the ST population for designing effective programmatic interventions. This study's primary aim is to analyse the extent of wealth-related inequalities in the undernutrition status among the children of the ST population and propose specific possible policy interventions for further improvement of their health status. In addition, this study also analyses the trends in the undernutrition status of children of the ST population between 2005 and 2021 in the two tribal-dominated states of India, namely Odisha and Jharkhand. Both Jharkhand and Odisha are among the Empowered Action Group (EAG) states which are tribal dominated. A significant proportion of the total population in Odisha (22·8%) and Jharkhand (26·2%) belong to the tribal category. The states constitute 8·3%, and 7·5% of the total ST population in India, respectively. Table 1 shows the socio-demographic profiles of both states.

**Table 1:** Jharkhand and Odisha: demographic and health indicators.

	India	Odisha	Jharkhand
% Rural population a	45.3	63.5	51.6
% Tribal population a	8.6	22.8	26.2
Literacy rate among STs a	59	52.2	57.1
Infant Mortality Rate b	30	38	27
Maternal Mortality Ratio b	7.3	9.7	5.6
% Institutional deliveries c	88.6	92.2	75.8
% Children aged under five who are stunted c	35.5	31.0	39.6
% Children (aged 12–23 months) Fully immunized c	76.4	90.5	73.9
% Households covered by improved sanitation facility c	70.2	60.5	56.7

a Census of India 2011, Office of the Registrar General, India.

b Sample Registration System of Registrar General of India.

c NFHS-5 fact sheet.

This paper is organised as follows: In section 2, we describe the data sources and methodology. In section 3.1, we present our analysis of the trends in the prevalence of stunting, underweight, and wasting among children under the age of five belonging to Scheduled Tribes in Odisha and Jharkhand between 2005 and 06 and 2019–21. To present the trends in undernutrition, we have categorised the population into three groups: STs, SCs and Others (including other backward castes and the general caste population). Scheduled castes are sub-communities within the framework of the Hindu caste system who have historically experienced deprivation, discrimination, and considerable social isolation in India on account of their supposed ‘low status’. These communities were notified as SC as per provisions contained in Clause 1 of Article 341 of the constitution. Both STs and SCs are the most disadvantaged socio-economic groups in India. Section 3.2 of this study analyses the trends in wealth-related inequalities in nutritional outcomes among children within ST communities in these states for the same period. Further, in section 3.2 we have also estimated the correlation between Antenatal Care and nutritional indicators. In 4, 5, we conclude with a brief discussion of the implications of this study and indicate ways to address the problem of undernutrition among the tribal children of Indian States.

## 2 Methodology

### 2.1 Data source

The study results are based on unit-level data from three rounds of the National Family Health Survey (NFHS), including NFHS-3 (2005–06), NFHS-4 (2015–16) and NFHS-5 (2019–21). NFHS is a large-scale, multi-round survey conducted by the Indian Institute of Population Sciences, Mumbai, in a representative sample of households throughout India. The survey captures national and state-level data on fertility, infant and child mortality, the practice of family planning, maternal and child health, reproductive health, nutritional status, utilization and quality of health and family planning services [23], [24]. The NFHS dataset is publicly available.

The NFHS provides data on standard anthropometric components for children under the age of five. Children who are less than two standard deviations (-2SD) from the median value for the reference population with respect to height-for-age (stunted), weight-for-height (wasting) and weight-for-age (underweight), whoare considered malnourished. The standard deviation score is also termed as Z-score and is the most widely used system for the analysis and presentation of anthropometric data [25].

The Z-score is calculated using the formula [26]:

$$Z\;\mathrm{score}\left(\mathrm{or}\;\mathrm{SD}\;\mathrm{score}\right)=\frac{\mathrm{observed value}-\mathrm{median value of the reference population}}{\mathrm{standard deviation value of the reference population}}$$

To estimate the inequality in nutritional outcomes among the ST community, we have used the data on the wealth quintile provided by NFHS. The wealth quintile in NFHS is approximated based on assets and household characteristics. The wealth scores are calculated using principal component analysis. The inequality is measured by dividing the wealth index into five quintiles (poorest, poorer, middle, richer and richest), with the lowest 20% representing the poorest and the highest quintile representing the richest 20% [23].

### 2.2 Statistical analysis

The study is a quantitative analysis using data from three rounds of the NFHS, a secondary data source. To compare and contrast the prevalence of undernutrition across different groups, we have categorized the population into three groups: Scheduled Tribes (ST), Scheduled Castes (SC) and others (Other backward castes and forward castes). We have estimated descriptive statistics of the nutritional status of the categorized social groups (the proportion of stunting, underweight and wasting). Further, we have analysed the trends in the nutritional status of ST, SC, and others versus all India population (including all the groups combined) across three time periods, NFHS-3 (2005–06), NFHS-4 (2015–16) and NFHS-5 (2019–21). We performed *t*-test to determine whether the differences in the mean between ST and Non-ST groups with respect to nutritional indicators are statistically significant or not. A Student's *t*-test is a ratio that quantifies how significant is the difference between the ‘means’ of two groups while considering their variance or distribution [27].

In the second part, we have estimated the existing wealth inequalities within ST communities using the Slope Inequality Index (SII) and Concentration Index (CIX) between 2005 and 2021. The SII is a logistic regression-based measure that calculates the difference in health outcomes between the highest and lowest wealth groups, while CIX is a relative measure of inequality. All the statistical analyses in the study are performed using Stata 15.0. The study employs equiplots to represent the gaps in nutritional status estimated among different wealth groups graphically. We have created equiplots with the help of *equiplots creator tool* developed by the International Centre for Equity in Health, Pelotas [28]. Further, to strengthen our study, we have analysed the correlation between Antenatal care and nutritional indicators using the Pearson Correlation test.

#### 2.2.1 Analysis of wealth-related inequalities

The SII measures the disparity in average health status between members of the highest and lowest socioeconomic groups (SEG). The simple linear regression model was first introduced by Preston, Haines, and Pamuk [29].

The SII is derived from the following simple linear regression model:

$$\mu =\beta_{0}+\beta_{1}R_{j}\;\mathrm{for}\;j=1-J$$

Where, *R*_*j*_ – Relative rank in the socio-economic group (SEG) distribution.

*β*_0_ – The estimated health status of a hypothetical person at the bottom of the hierarchy (*R*_*j*_ = 0).

Assuming linearity, SII is the difference between the average health status of the person at the bottom of the SEG hierarchy and the person at the top. As SII is calculated for grouped data, weighted least squares are employed, with equal weights assigned to the population of group *j* [29]. The formula for SII is given as:

$$\mathrm{SII}=\beta_{1}=\frac{\sum_{j=1}^{J}p_{j}R_{j}\left(\mu_{i}-\mu \right)}{\sum_{j=1}^{J}p_{j}R_{j}^{2}-{\left(\sum_{j=1}^{J}p_{j}R_{j}\right)}^{2}}$$

Where, *μ*_*j*_ – Average health status of SEG *j*

*p*_*j*_ – Population share of SEG *j*

*R*_*j*_ = ∑_*γ*_^*j*−1^*p*_*γ*−0.5*p*_*j*__ – Relative rank of SEG *j*

*μ* = ∑_*j*=1_^*J*^*p*_*j*_*μ*_*j*_ – Average health status of the population

The CIX measures relative inequality, similar to the Gini Index, which determines wealth/income inequality. It assesses the degree to which a specific socioeconomic grouping (SEG) has a higher prevalence of health or sickness than the general population. On the x-axis, individuals are ranked according to their socio-economic status, while the intervention coverage or prevalence is plotted on the y-axis. The concentration of intervention coverage or prevalence is measured by calculating the distance between the curve and the diagonal.

The Relative Concentration Index is defined as [30]:

$$\mathrm{CII}=\frac{2}{\mathrm{N\mu}}\left[\sum_{i=1}^{N}y_{i}R_{i}\right]-1$$

Where, μ - Population mean of Y

*R*_*i*_ – Relative rank of socioeconomic status for the i^th^ individual

*y*_*i*_ – Health Outcome

N – Population Size

The SII and CIX values vary between −100 to +100 percentage points [31]. A positive value shows a pro-rich pattern with a higher prevalence of undernutrition among children from rich households. A negative value, on the contrary, implies a pro-poor pattern with the concentration of undernutrition among children from poor households. Zero represents perfect equality i.e. the nutritional outcome is identical across all the wealth quintiles.

## 3 Results

### 3.1 Trends in prevalence of undernutrition among children belonging to ST, SC and other households under 5 in Odisha and Jharkhand

**Fig. 1:**
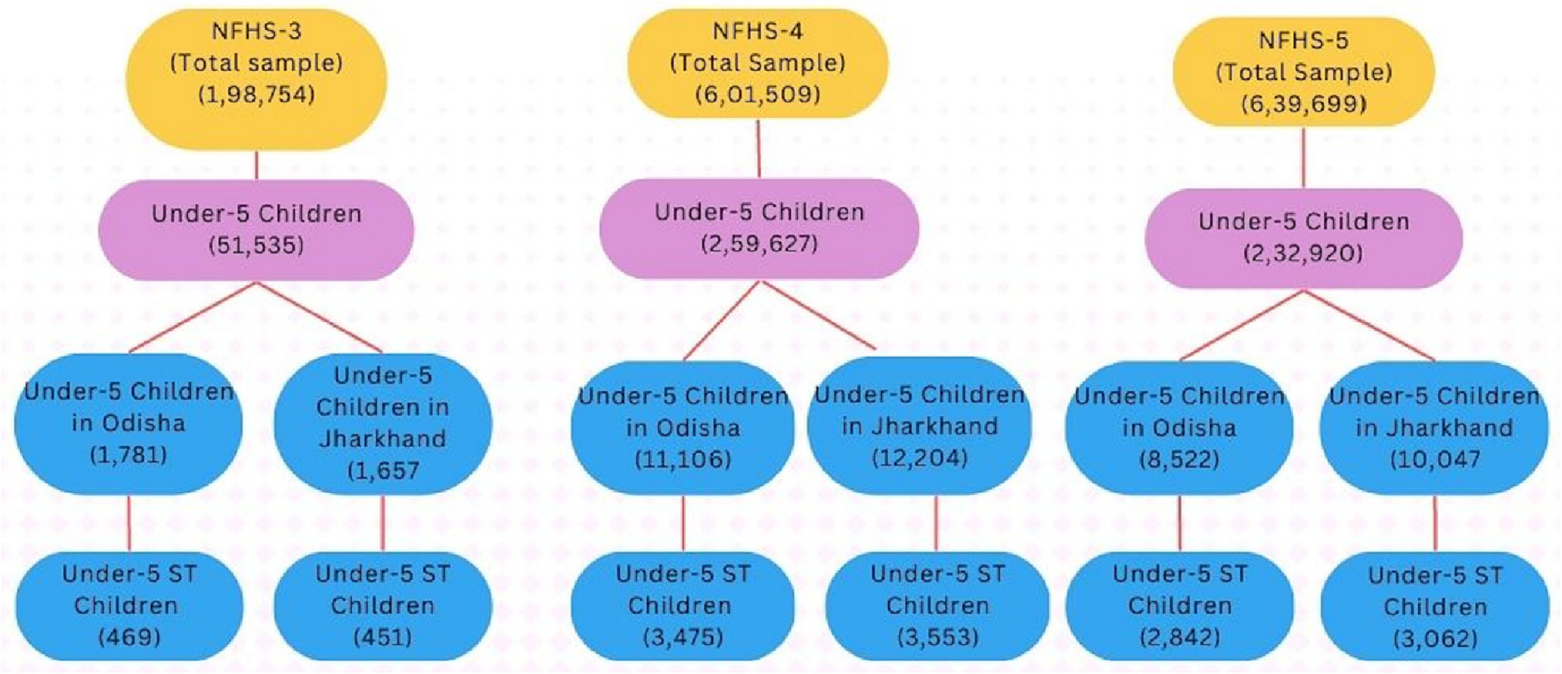
The sample size of under-5 children across three rounds of NFHS at All India level, Jharkhand and Odisha.

**Fig. 2:**
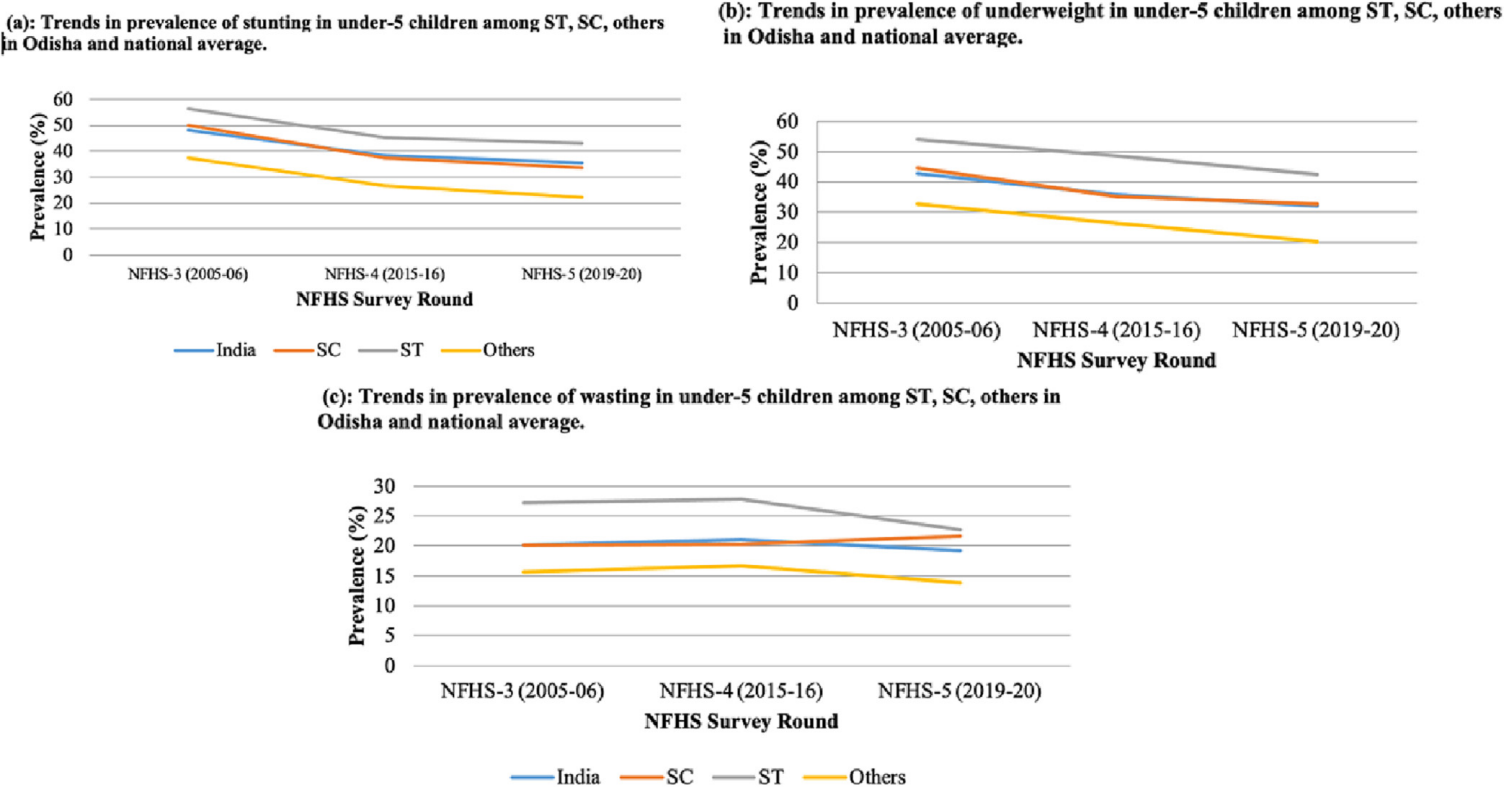
Trends in the prevalence of undernutrition among under-5 children belonging to ST, SC and other households in Odisha.

**Fig. 3:**
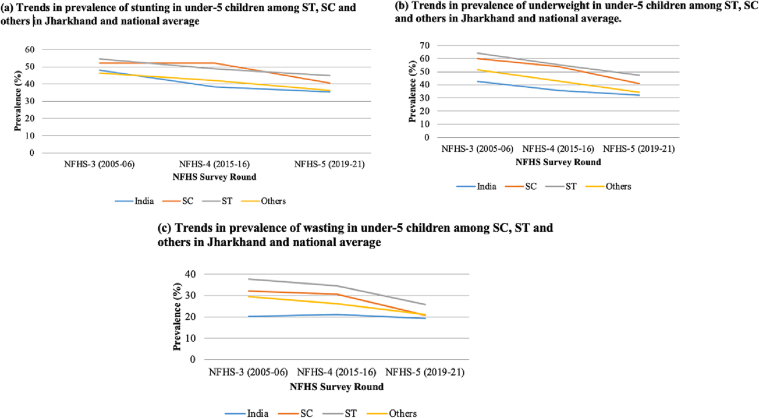
Trends in prevalence of undernutrition among under-5 children belonging to ST, SC and other households in Jharkhand.

**Fig. 4:**
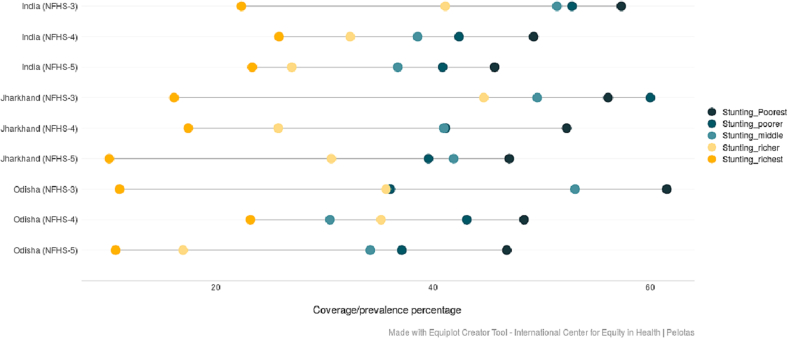
Trends in the prevalence of stunting among under-5 children across wealth quintiles belonging to ST community in India, Jharkhand and Odisha (NFHS-3 (2005–06), NFHS-4 (2015–16) and NFHS-5 (2019–21).

**Fig. 5:**
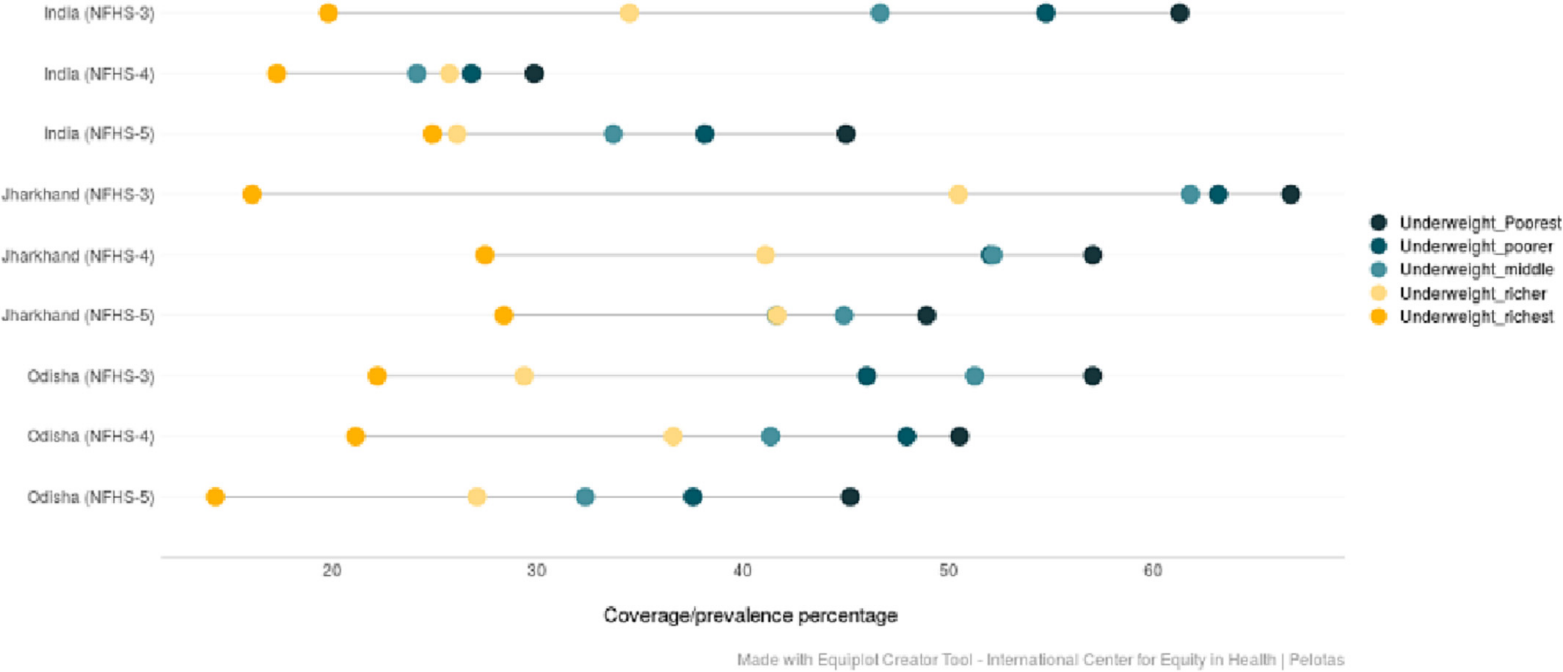
Trends in the prevalence of underweight among under-5 children across wealth quintiles belonging to ST community in India, Jharkhand and Odisha (NFHS-3 (2005–06), NFHS-4 (2015–16) and NFHS-5 (2019–21)).

**Fig. 6:**
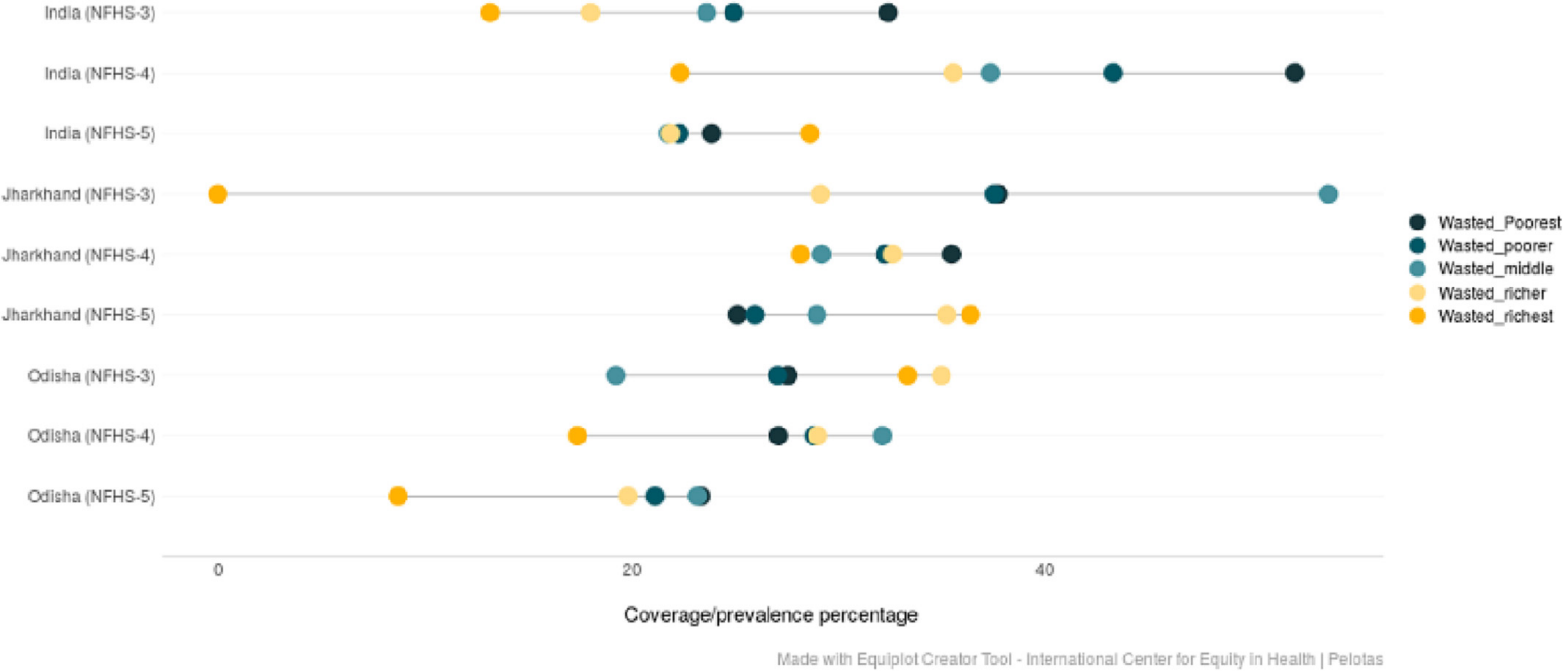
Trends in the prevalence of wasting among under-5 children across wealth quintiles belonging to ST community in India, Jharkhand and Odisha (NFHS-3 (2005–06), NFHS-4 (2015–16) and NFHS-5 (2019–21)).

In this section, we have compared the trends in the prevalence of stunting, underweight and wasting among children belonging to different social groups in Odisha and Jharkhand and all-India, between three rounds of the NFHS survey (Fig. 1, Fig. 2, Fig. 3 for Odisha; Fig. 4, Fig. 5, Fig. 6 for Jharkhand). Further, we have analyzed the trends in the wealth-related inequalities (absolute and relative) among ST children in Odisha and Jharkhand. The disparities in the nutritional status among ST children belonging to different wealth quintiles – poorest (Q1), poorer (Q2), middle (Q3), richer (Q4) and richest (Q5) in Odisha and Jharkhand and at the national level are presented employing equiplots.

(Source: Authors' estimates based on unit level data of NFHS-3, NFHS-4 and NFHS-5).

(Source: Authors' estimates based on unit level data of NFHS-3, NFHS-4 and NFHS-5).

(Source: Authors' estimates based on unit level data of NFHS-3, NFHS-4 and NFHS-5).

(Source: Authors' estimates based on unit level data of NFHS-3, NFHS-4 and NFHS-5).

(Source: Authors' estimates based on unit level data of NFHS-3, NFHS-4 and NFHS-5).

The prevalence of stunting in Odisha has reduced by 13 percentage points (pp), 17 pp. and 4 pp. among ST, SC and others, respectively, between NFHS-3 (2005–06) and NFHS-5 (2019–21). There is an increase in the gap in the prevalence of stunting among ST when compared to SC and others between the third and the fifth rounds of the survey (Fig. 2a). This indicates that the prevalence of stunting has reduced more among SC and others than in ST populations in the past 15 years.

There is a reduction in the prevalence of underweight among ST, SC and others, indicating an improvement in the nutrition status among under-5 children. The percentage fall in underweight is about 6 pp. among ST children in Odisha between NFHS-3 (2005–06) and NFHS-5 (2019–21). Moreover, among the SC and others, we have observed a reduction of 12 pp. each in the same period (Fig. 2b). The prevalence of wasting showed an increase among the SC (2 pp) between the third and fifth rounds but exhibited a reduction in the past 15 years for ST, others (2 pp) and at the national level (1 pp). A fall of 4 pp. is observed in wasting among ST children between three rounds of NFHS (Fig. 2c).

The proportion of stunted, underweight and wasted under-5 children has decreased for ST, SC and others in the state of Jharkhand between the third and fifth rounds of NFHS surveys. The percentage fall in stunting, underweight and wasting among ST children is 10 pp., 17 pp. and 12 pp., respectively, between NFHS-3 (2005–06) and NFHS-5 (2019–20). The prevalence of stunting, underweight and wasting decreased by 12 pp., 19 pp. and 12 pp. among SC children and 10 pp., 17 pp. and 8 pp. among others, respectively, between NFHS-3 (2005–06) and NFHS-5 (2015–16) (Fig. 3). The proportion of stunting is found to be higher among children belonging to SC communities when compared to ST and others in the fourth round of NFHS (2015–16). Similar to Odisha, Jharkhand exhibits an improvement in the nutritional outcomes among children belonging to all social groups.

We conducted a *t*-test to determine if the differences in the means between ST and Non-ST groups concerning stunting, underweight and wasting in India are significant. The results indicate that differences in the mean values of the nutritional indicators are statistically significant and positive (Table 2). The results of the *t*-test imply that there is an improvement in the nutrition outcomes among the ST population, and hence the mean difference between ST and non-ST populations has reduced over the years.

**Table 2:** Differences in mean (t-test) Height-for-age z-scores, weight-for-age z-scores and weight-for-height z-scores between the ST and non-ST group.

	Stunting (Height-for-age)		Underweight (Weight-for-age)		Wasting (Weight-for-height)	
	MD	SE	MD	SE	MD	SE
India						
NFHS-3	489·5***	12·5	495·1***	12·4	500·5***	12·3
NFHS-4	100·19***	5·03	106·06***	5·03	111·36***	5
NFHS-5	16·1*	3·89	13·61***	4·28	−16·91	5·99

MD: Mean Difference.

SE, Standard Error.

P value: * p < 0·10, ** p < 0·05, *** p < 0·01.

Source: Authors' estimates based on unit level data of NFHS-3, NFHS-4 and NFHS-5.

### 3.2 Trends in wealth related inequalities in nutrition status among children belonging to ST households in Jharkhand and Odisha

The equiplots (Fig. 4, Fig. 5, Fig. 6) show the prevalence of stunting, wasting and underweight among ST children across wealth quintiles in India and the two states in the last 15 years. The wealth-related inequality in undernutrition outcomes in Odisha and Jharkhand among the ST children is assessed using SII and CIX (Table 2 and Table 3).

**Table 3:** Trends in wealth-related absolute (SII) and relative (CIX) inequalities in nutritional outcomes among ST children in Odisha.

SII	NFHS-3 (2005–06)	NFHS-4 (2015–16)	NFHS-5 (2019–21)	Change in absolute inequality between NFHS-3 and NFHS-5^
Odisha				
Stunting	−0·41*** (−0.59, −0.23)	−0·19*** (−0.26, −0.12)	−0·27*** (−0.35, −0.19)	−0·14
Underweight	−0·27*** (−0.47, −0.07)	−0·12*** (−0.20, −0.05)	−0·21** (−0.28, −0.13)	−0·06
Wasting	−0·02 (−0.21, 0.16)	0·014 (−0.04, 0.07)	−0·05 (−0.11, 0.01)	0·03


CIX	NFHS-3 (2005–06)	NFHS-4 (2015–16)	NFHS-5 (2019–21)	Change in relative inequality between NFHS-3 and NFHS-5^
Odisha				
Stunting	−0·077*** (−0.12,−0.02)	−0·052*** (−0.07, −0.03)	−0·049*** (−0.07, −0.02)	−0·028
Underweight	−0·073*** (−0.12, −0.02)	−0·031*** (−0.05, −0.009)	−0·038*** (−0.06, −0.01)	−0·035
Wasting	−0·02 (−0.11, 0.07)	−0·003 (−0.03, 0.02)	−0·045 (−0.08, −0.004)	0·025

Source: Authors' estimates based on unit level data of NFHS-3, NFHS-4 and NFHS-5.

*P* value: * *p* < 0·10, ** *p* < 0·05, *** p < 0·01.

^Note: ‘(−) negative sign)’ signifies a reduction in SII values from 2005 to 06 to 2019–21.

The equiplots show that there is a reduction in the proportion of stunted children belonging to both poor and rich households in India, Odisha and Jharkhand between NFHS-3 (2005–06) and NFHS-5 (2019–21) (Fig. 4). For underweight, although there has been a visible reduction in ST children belonging to lower wealth quintiles, the proportion among children from higher wealth quintiles have increased in India and Jharkhand between NFHS-3 (2005–06) and NFHS-5 (2019–21) (Fig. 5). The prevalence of wasting declined among the ST children from poor households in India and both the states between third and fifth rounds of NFHS. The proportion of wasted children is higher among ST households belonging to the highest wealth quintile in NFHS-5 (2019–21) at the national level as well as in Jharkhand (Fig. 6).

Table 3 shows the wealth-related inequalities (absolute and relative) in the prevalence of nutrition outcomes among ST children in Odisha. The overall trends in wealth-related inequalities with regards to stunting as well as underweight show that the prevalence of these two indicators of undernutrition is concentrated among poorer ST children. While considering stunting in Odisha, there is a 22 pp. fall in the absolute inequality and 2.5 pp. in relative inequality between NFHS-3(2005–06) and 4(2015–16). However, an increase of 8 pp. in absolute inequality and a reduction of 0.3 pp. in relative inequality is observed between NFHS-4 (2015–16) and 5 (2019–21). In the case of the prevalence in underweight, a reduction in the absolute (15 pp), as well as relative inequalities (4.2 pp), is evidenced between NFHS-3(2005–06) and 4 (2015–16). This is followed by an increase of 9 pp. and 7 pp. in absolute and relative inequalities, respectively between NFHS-4 (2015–16) and 5 (2019–21) in Odisha. The wealth-related inequalities for wasting are found to be insignificant across all three rounds of NFHS in Odisha (Table 3). As we can see from Table 3, there is a reduction in the absolute as well as relative inequalities with respect to stunting and underweight among under-5 ST children between NFHS-3 (2005–06) and 5 (2019–21) in Odisha.

Table 4 shows the wealth-related inequalities (absolute and relative) in the prevalence of nutrition outcomes among ST children in Jharkhand. The absolute, as well as relative inequalities for stunting have increased in Jharkhand by 25 pp. and 0.7 pp. respectively, between NFHS-3(2005–06) and 4 (2015–16). However, there is a reduction in the inequalities (both absolute and relative) between NFHS-3(2005–06) and 5 (2019–21). Despite the recent reduction, the prevalence of stunting is still higher among ST children of poorer households. There is a decline in the absolute inequality in the prevalence of underweight by 11 pp. between NFHS-3(2005–06) and 5 (2019–21), but during the same period, there is only a 1 pp. reduction in relative inequality for underweight. As observed in Odisha, in Jharkhand too the wealth-related inequalities for wasting are found to be insignificant across all three rounds of NFHS.

**Table 4:** Trends in wealth-related absolute (SII) and relative inequalities (CIX) in nutritional outcomes among ST children in Jharkhand.

SII	NFHS-3 (2005–06)	NFHS-4 (2015–16)	NFHS-5 (2019–21)	Change in absolute inequality between NFHS-3 and NFHS-5^
Jharkhand				
Stunting	−0·2* (−0.42, −0.007)	−0·27*** (−0.35, −0.19)	−0·18*** (−0.27, −0.10)	−0·02
Underweight	−0·26*** (−0.42, −0.07)	−0·15*** (−0.23, −0.07)	−0·15*** (−0.24, −0.06)	−0·11
Wasting	−0·038 (−0.25, 0.18)	−0·06 (−0.13, 0.01)	−0·003 (−0.07, 0.08)	−0·035

CIX	NFHS-3 (2005–06)	NFHS-4 (2015–16)	NFHS-5 (2019–21)	Change in relative inequality between NFHS-3 and NFHS-5^
Jharkhand				
Stunting	−0·05** (−0.1, −0.02)	−0·057*** (−0.07, −0.01)	−0·03*** (−0.05, −0.007)	−0·02
Underweight	−0·03* (−0.07, −0.01)	0.0·020*** (−0.03, −0.001)	−0·029*** (−0.05, −0.006)	−0·001
Wasting	0·015 (−0.06, 0.09)	−0·004 (−0.03, 0.02)	−0·013 (−0.02, 0.05)	0·002

Source: Authors' estimates based on unit level data of NFHS-3, NFHS-4 and NFHS-5.

*P* value: * *p* < 0·10, ** p < 0·05, *** p < 0·01.

^Note: ‘(−) negative sign signifies a reduction in CIX values between 2005 and 06 to 2019–21.

The overall trends show that stunting, underweight and wasting are still concentrated among ST children belonging to poor households in Jharkhand and Odisha. The pattern of absolute and relative inequality over the years does not show a uniform increase or decrease over the years in both states.

### 3.3 Correlation between ANC visits and nutritional indicators. Inequality in the utilization of ANC services

Table 5 shows the correlation between ANC visits and undernutrition indicators (stunting, underweight and wasting). We observed a negative (statistically significant) correlation between ANC visits and undernutrition indicators. The higher the number of ANC visits among women, the lower the prevalence of stunting, underweight and wasting among children.

**Table 5:** Correlation of ANC visits and malnutrition outcomes.

	ANC visits	Stunting	Underweight	Wasting
ANC visits	1.0000			
Stunting	−0.0107*	1.0000		
Underweight	−0.0154*	0.3971*	1.0000	
Wasting	−0.0241*	−0.0966*	−0.3668*	1.0000

Significant at *p* < 0.001.

Source: Authors' estimates from NFHS-5 unit level data.

We found a relatively lower coverage of ANC visits among ST as compared to forward castes (Table 6). In addition, we observed a huge within-group disparity with respect to the proportion of ST women attending four or more ANC visits. For instance, in Jharkhand, the coverage among ST women belonging to the poorest households was only 30% against 73% among women from the richest households (Table 7).

**Table 6:** Proportion of women attending ANC visits (4 or more) across social groups (Scheduled tribes; ST vs forward caste) (NFHS-5;2019–21).

Wealth quintile to which the ST women belongs to	All India (%)	Jharkhand (%)	Odisha (%)
Forward caste^1^	63	47	81
ST	56	37.2	70
Difference (in percentage point)	7	9.8	11

Source: Authors' estimates from NFHS-5 unit level data.

1 Forward caste includes ‘others’ category according to NFHS-5.

**Table 7:** Wealth quintile wise proportion of women attending ANC (4 or more) within the ST group (NFHS-5; 2019–21):

Wealth quintile to which the ST women belongs to	All India (%)	Jharkhand (%)	Odisha (%)
Richest	74	73	84
Poorest	51	30	67
Difference (in percentage point)	17	43	17

Source: Authors' estimates from NFHS-5 unit level data.

## 4 Discussion

Despite the overall improvement and the advances in the health status of the country's population, the tribal communities of India remain highly vulnerable owing to food insecurity, under-nutrition and ill health [32,33]. Our study based on the unit data from NFHS (over the past 15 years) reinforces this observation.

The findings in this study provide insights into the trends in the prevalence of stunting, underweight and wasting among the tribal population in the two tribal-dominated states of India, Odisha and Jharkhand. Although the trends exhibit an improvement in the nutritional status over the years among children belonging to all the social groups in India, the nutrition status of children belonging to the ST population lags behind when compared to others.

Stunting, which is indicative of chronic undernutrition, is mainly prevalent in less developed states. Whereas the prevalence of wasting, an indicator of acute undernutrition, is principally seen among children from more developed states. Evidence suggests that it is difficult to bring a simultaneous reduction in both stunting and wasting, as a decline in stunting is accompanied by an increase (or no improvement) in wasting [33] This is visible in the context of India in the results of the current study. In India, between NFHS-3 (2005–06) and NFHS-5 (2019–21), stunting has reduced by 26% at the national level, whereas wasting has reduced by a mere 4%. Similarly, we observe a reduction in the prevalence of stunting at the national level as well as in both states, accompanied by an increase in wasting between NFHS-3 (2005–06) and NFHS-4 (2015–16),.

In India, the persisting economic inequalities have led to a larger concentration of anaemia, stunting, wasting and underweight among the children belonging to the poorest wealth quintile. Wealth-related inequalities in the nutrition outcomes among children have been studied at the national as well as subnational levels. Similar results are observed in other scholarships as well. In an attempt to measure the existing socio-economic inequalities in chronic childhood malnutrition, it is discerned that there is a disproportionate burden of stunting among children from poor socio-economic positions [11,28,34] Our analysis also shows significant wealth-related inequalities in the prevalence of stunting and underweight (except wasting) among ST children of the tribal-dominated states of Odisha and Jharkhand. The trend shows a reduction in wealth-related absolute as well relative inequalities in stunting and underweight in Odisha and Jharkhand between NFHS-3 (2005–06) and NFHS-5 (2019–21). The SII and CIX values for wasting do not show a significant association between wealth and wasting, indicating that wasting might be prevalent among children of all the wealth groups in both Jharkhand and Odisha. Despite the decline in the within-group inequalities in child nutrition outcomes over the years, a child from the lowest wealth quintile within the ST community is the most disadvantaged. It can be attributed to their poor access to health resources as well as because of their social and economic position. [35,17]. Governments have been implementing several programmatic interventions to improve the overall nutritional status of children across India. They include Integrated Child Development Services (ICDS), National Rural Health Mission including Janani Suraksha Yojana, the Total Sanitation Campaign, National Rural Drinking Water Programme, Mid-Day Meals Scheme, Public Distribution System, National Food Security Mission and National Rural Livelihoods Mission [9]. These programmes aim to effectively address the broader determinants of undernutrition across the life cycle of each individual. ICDS is a flagship programme of the Government of India that provides health and nutrition services to children below 6 years, adolescent girls and pregnant women through a network of village-level centres. Studies have found the supplementary nutrition services by ICDS to be an effective intervention in the improvement of child nutrition outcomes [36,37]. A revamped National Nutritional Mission (*POSHAN Abhiyan*) with a 1·3 billion USD budget was launched in 2018 by the Government of India to address the issue of persistent undernutrition in the nation. The programme concentrates on improving the nutritional status of tribal children in particular [33,38].

We suggest the policymakers to strengthen and focus on the following components, which could positively affect the nutritional status of ST children:

One of the ways is to diversify the diets of the ST population by including millet as it is anefficient and cost-effective method. A study jointly conducted by ICRISAT, UNICEF, NIN found that a diversified diet that includes millet improves the height and weight of children in regions where rice is the staple food. Hence, diversification of standard diets using millet could be an effective step towards reducing malnutrition [39]. Also, there is a need to improve access to health care services such as supplementary nutrition programme by ICDS, preconception care of pregnant women and newborn care at an earlier stage. It has been found that caste barriers can adversely affect the uptake of ICDS services. Hence, an increase in the representation of ICDS workers belonging to SC/ST groups may help to improve the uptake of the services among the beneficiaries from these communities [40]

Further, disparities in access to services forpregnant women may affect child nutrition outcomes. As found in our study and some previous studies that there is a significant negative association between mother's ANC visits and child's undernourishment status [38,41] The utilization of ANC can be improved by the inclusion of local stakeholders, such as tribal healers and local dais in providing essential health education to mothers. Stakeholders can effectively communicate the importance of early initiation of breastfeeding, exclusive breastfeeding, full ANC, and timely initiation of complementary feeding and immunization to the beneficiaries. To overcome the geographical barrier to access among ST women Government of Tamil Nadu introduced ‘birth waiting rooms’ in 17 tribal hamlets, where the pregnant women are admitted two weeks before the delivery to provide safe institutional delivery [42]. This can be extended to different states where accessibility is difficult inorder to improve institutional deliveries. Our analysis of the nutrition status among children of the ST population in Odisha and Jharkhand demonstrates the urgency to have a policy focus on inequities within the tribal population of India.

## 5 Conclusion

Our study results show that, although the trend exhibits an overall improvement in the nutritional status in India over the years, the tribal children are yet to catch up with their non-tribal counterparts, and the ST children belonging to the poorest households are yet to catch up with the ST children belonging to rich households.

The results of our study corroborate with the existing literature on undernutrition among tribal children in the country. Our analysis shows a statistically significant reduction in wealth-related inequalities in the prevalence of stunting and underweight among ST children of the tribal population in Jharkhand and Odisha. We did not find any wealth-related inequalities with respect to wasting among the under-5 tribal children in the two states.

The study's main strength is that it uses multiple rounds of large-scale data to estimate nutritional disparities among the under-five children of ST population. The analysis is strengthened further by the use of the Slope Inequality Index and Concentration Index to quantify the degree of inequality within ST communities.

The use of secondary data alone is not sufficient to shed light on the reasons for the prevalence of inequality in malnutrition status. The contextual factors that precipitate malnutrition among children under age five could be unique to each tribal region. A primary survey on the underlying factors would provide a better understanding of the prevalence of undernutrition among the children of the ST population.

## Availability of data and materials

The datasets generated during and/or analysed in the current study are available on the DHS and IIPS website, accessed from https://dhsprogram.com/data/.

## Declaration of Competing Interest

The authors declare that they have no known competing financial interests or personal relationships that could have appeared to influence the work reported in this paper.
